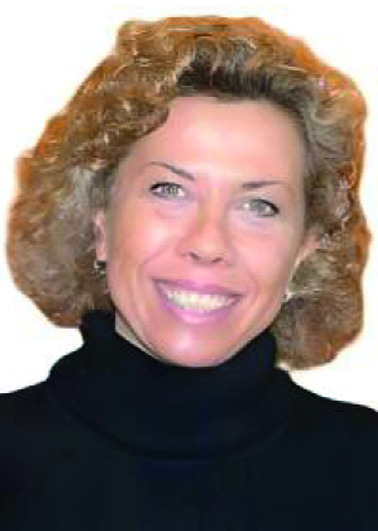# Advances and Challenges in the Development of Immobilized Enzymes for Batch and Flow Biocatalyzed Processes

**DOI:** 10.1002/cssc.202402007

**Published:** 2024-12-03

**Authors:** Stefania Patti, Ilaria Magrini Alunno, Sara Pedroni, Sergio Riva, Erica Elisa Ferrandi, Daniela Monti

**Affiliations:** ^1^ Istituto di Scienze e Tecnologie Chimiche “G. Natta” (SCITEC) CNR Via Bianco 9 20131 Milano Italy; ^2^ Department of Pharmaceutical Sciences University of Milan Via Mangiagalli 25 20133 Milano Italy

**Keywords:** Biocatalysis, Enzyme immobilization, Carrier materials, Batch and flow processes, Genetic fusions

## Abstract

The development of immobilized enzymes both for batch and continuous flow biocatalytic processes has gained significant traction in recent years, driven by the need for cost‐effective and sustainable production methods in the fine chemicals and pharmaceutical industries. Enzyme immobilization not only enables the recycling of biocatalysts but also streamlines downstream processing, significantly reducing the cost and environmental impact of biotransformations. This review explores recent advancements in enzyme immobilization techniques, covering both carrier‐free methods, entrapment strategies and support‐based approaches. At this regard, the selection of suitable materials for enzyme immobilization is examined, highlighting the advantages and challenges associated with inorganic, natural, and synthetic organic carriers. Novel opportunities coming from innovative binding strategies, such as genetic fusion technologies, for the preparation of heterogeneous biocatalysts with enhanced activity and stability will be discussed as well. This review underscores the need for ongoing research to address current limitations and optimize immobilization strategies for industrial applications.

## Graphical abstract

Recent advancements in the preparation of immobilized enzymes have improved both batch and continuous flow biocatalytic processes, essential for cost‐effective, sustainable production in the fine chemicals and pharmaceutical industries. This review examines advancements in carrier‐free methods, entrapment strategies, and support‐based approaches, discusses material choices, and explores novel binding techniques like genetic fusion for better enzyme activity and stability.

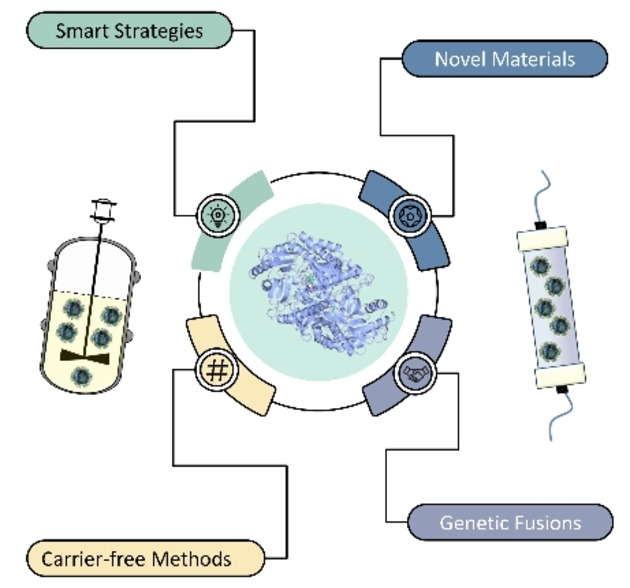

## 1 Introduction

With an increasing focus on utilizing biocatalysis in fine chemicals and pharmaceutical industry, various complementary technologies have emerged to facilitate the advancement of intensified and large‐scale biocatalytic processes.^[1]^ This includes the discovery and optimization of highly efficient enzymes, easily available as recombinant proteins, innovative retrosynthetic approaches emphasizing the integration of biocatalytic steps into existing production pathways, and strategies for enzyme recycling/recovery. Collectively, these advancements contribute to enhancing the overall prospects of biocatalysis and its industrial application.[^2^, ^3^]

Enzyme immobilization plays a central role in both biocatalyst recycling and straightforward downstream processing, thus favoring the reduction of biotransformation costs and environmental impact toward an overall improved sustainability. For these reasons, this topic has been investigated since the early days of biocatalysis for the application of enzymes in industry,[^4^, ^5^, ^6^, ^7^] as well as for the development of enzyme‐based biosensors.[^8^, ^9^]

However, advancements in bioprocess and protein engineering, as well as in material science, are continuously unveiling fresh prospects in this field for the future. As a result, enzyme immobilization, still far from achieving maturity,^[10]^ remains a highly intriguing area, requiring substantial research endeavors to fully maximize its potential.

Moreover, of note is the rising interest in conducting enzyme‐catalyzed transformations in continuous flow systems, which may offer several advantages over batch processes, such as enhanced reaction productivity and scalability and the possible coupling to tailored (by)product(s) removal and downstream processing steps. On this respect, it might be foreseen that modular and easily interchangeable reactors could be designed and used sequentially or in cascades according to the specific needs.[^11^, ^12^] Advances in the development of innovative batch reactors, such as for example rotating bed reactors,^[13]^ that could permit scalable process intensification along with biocatalysts reuse, are highly foreseen as well.

A variety of techniques are available for enzyme immobilization; however, the industry predominantly prefers methods that are both simple and cost‐effective. Among these, physical immobilization methods, that involve non‐covalent interactions to attach enzymes to a support matrix (such as adsorption and physical entrapment), and chemical immobilization methods (such as covalent binding and cross‐linking) are the most commonly employed.

**Figure 1:**
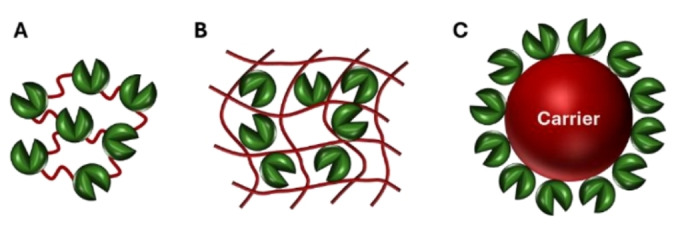
Main strategies for enzyme immobilization. A) Cross‐linking of enzyme molecules; B) entrapment of enzyme molecules in hydrogel or polymeric matrices; C) binding to an insoluble carrier.

Specifically, there are three primary techniques for enzyme immobilization that are currently used in biocatalyzed batch or flow processes: (i) self‐immobilization without a carrier through the cross‐linking of enzymes, (ii) trapping them within a carrier such as a hydrogel or polymer matrix that forms in the presence of the enzyme, (iii) attaching them to an insoluble organic or inorganic carrier (Figure 1). All three methods transform a free enzyme from a water‐soluble homogeneous catalyst into a solid heterogeneous catalyst. Alternatively, a compartmentalized system can be set up, where the enzyme is dissolved in the reaction medium, but confined into the reactor by a membrane, which allows for substrate and/or product permeability in either fed‐batch or continuous biocatalytic processes.

In this review, we will present recent progress in the design and preparation of immobilized enzymes that are suitable as heterogeneous biocatalysts for batch and flow processes. Strategies based on the cross‐linking and entrapment of enzyme molecules will be treated first, then methodologies based on enzyme binding to a carrier will be discussed. At this regard, a specific focus will be on the selection of suitable materials and binding modes, from simple adsorption to sophisticated genetic fusions.

## 2 An Update on the Use of Carrier‐Free and Entrapment‐Based Immobilization Approaches

### 2.1 Developments in the Application of Carrier‐Free Strategies for Enzyme Immobilization

Carrier‐free immobilization refers to methods of enzyme immobilization that do not rely on an external solid support or carrier material. These methods involve cross‐linking of enzymes and their derivatives to form insoluble and catalytically active particles. This approach is cost‐effective as it eliminates the expense associated with using carriers and it avoids issues related to the carrier′s physicochemical properties affecting enzymatic activity. In addition, using carriers for enzyme immobilization typically results in significant dilution of the biocatalyst within the reaction medium, as the carrier occupies about 95 % of the carrier‐enzyme volume, and this often leads to low space‐time yields and catalytic efficiency. In contrast, carrier‐free immobilization allows, in principle, for high productivity, measured as kilograms of product per kilogram of biocatalyst, and high space‐time yields.^[14]^

To date, the most convenient and widely used method in this field is the self‐immobilization of enzymes through the formation of cross‐linked enzyme aggregates (CLEAs). The formation of CLEAs involves a two‐step process: first, an enzyme precipitation, typically obtained by adding an inorganic salt such as ammonium sulfate, followed by the addition of a bifunctional reagent to cross‐link the aggregates, making them permanently insoluble while preserving their pre‐organized tertiary structure and activity.[^7^, ^15^] Glutaraldehyde is certainly the most commonly used cross‐linker as it is economical and highly effective for CLEAs formation. However, it has some drawbacks, including a recognized environmental toxicity, a frequent detrimental effect on enzyme activity by direct interaction with active site residues and its relatively short linker length that can results in very compact CLEAs, showing possible mass transfer limitations. Alternatively, other cross‐linkers have been used, such as macromolecular cross‐linkers derived from readily available polysaccharides, like pectin,^[16]^ the convenient and inexpensive bisepoxide glycerol diglycidyl ether that could confer an improved mechanical stability to CLEAs,^[17]^ or polymers containing amino‐groups such as polyethylenimines.^[18]^

CLEAs are insoluble and therefore they can be recovered for recycling through centrifugation, filtration, or decantation, thereby simplifying downstream processing. Notably, CLEAs recovery can be further enhanced, eliminating the need for separation steps like centrifugation or filtration, through the formation of magnetic CLEAs (m‐CLEAs). In fact, enzymes can be cross‐linked in the presence of amino‐functionalized Fe_3_O_4_ particles, resulting in m‐CLEAs that can be swiftly and efficiently retrieved using a magnet.^[7]^ For instance, m‐CLEAs of L‐arabinose isomerase were obtained using magnetic nanoparticles functionalized with 3‐aminopropyltriethoxysilane and successfully employed for the isomerization of D‐galactose to D‐tagatose, a functional sweetener, then recycled several times with little loss of activity.^[19]^ A systematic comparison of the performance of CLEAs and m‐CLEAs of the cellulase from *Trichoderma reesei* was recently carried out.^[20]^ Both enzyme preparations showed higher activities in comparison with the activity of the free enzyme and were efficiently re‐used for up to 10 cycles with almost comparable operative stability.

Other advantages of CLEAs are related to their enhanced storage and operational stability, showing high resistance to denaturation by heat, organic solvents, and autolysis. They are also highly stable against leaching in aqueous media and under high ionic strength conditions and they can be utilized in both aqueous media and organic solvents.

Most recent examples related to CLEAs involve the use of the so‐called “combi‐CLEAs”, where two or more enzymes are co‐immobilized to minimize the diffusion of intermediates in the reaction medium while still allowing substrates and coenzymes to access the active sites.^[21]^

Combi‐CLEAs of cyclodextrin glucanotransferase and maltogenic amylase from *Bacillus lehensis* G1 have been recently prepared to catalyze a reaction cascade aimed at the obtainment of maltooligosaccharides (MOS) from soluble starch.^[22]^ MOS are commonly used in the food industry as sweeteners, bulking agents, and as a source of easily digestible carbohydrates and prebiotics. In this work, combi‐CLEAs were obtained using chitosan or *O*‐carboxymethyl chitosan as cross‐linkers. The introduction of longer side chains of carboxymethyl group resulted in a more flexible structure of combi‐CLEAs leading to a higher catalytic efficiency and stability of the immobilized enzymes.

In another work, Vernet *et al*. catalyzed a linear cascade with *in situ* cofactor regeneration to synthesize ε‐caprolactone from cyclohexanol using the alcohol dehydrogenase (ADH) from *Thermoanaerobacter brockii* and the cyclohexanone monooxygenase (CHMO) from *Thermocrispum municipale*.^[23]^ In this cascade, cyclohexanol is oxidized to cyclohexanone by ADH, which simultaneously catalyzes the reduction of NADP^+^ to NADPH. Subsequently, CHMO oxidizes cyclohexanone to ε‐caprolactone and NADPH to NADP^+^ at the expense of molecular oxygen, making NADP^+^ readily available for the first step. The performance of CLEAs of a chimeric protein ADH‐CHMO (fusion‐CLEAs), combi‐CLEAs of single ADH and CHMO (co‐immobilization), and multi‐CLEAs (CLEA‐ADH and CLEA‐CHMO mix), prepared starting from recombinant *E. coli* cell‐free extract as the enzymatic source and using glutaraldehyde as the cross‐linker, were compared. These three CLEAs performed similarly, although the conversions were less satisfactory compared to those obtained with the free enzymes. Notably, the CLEAs of the chimeric protein were also used in the same reaction in microaqueous organic media and demonstrated promising operational and storage stability.^[23]^

**Figure 2:**
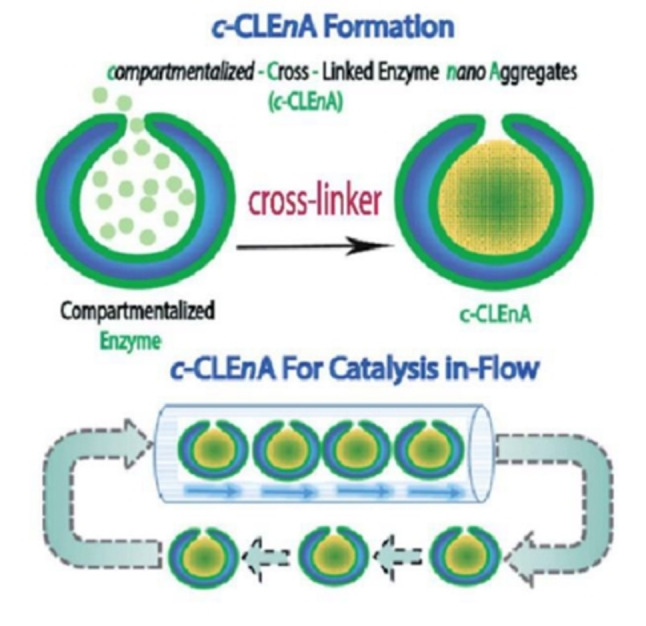
Preparation of compartmentalized‐cross‐linked enzyme nano‐aggregates (c‐CLEnA) and their application in flow biocatalysis. Reproduced from,^[27]^ copyright De Martino *et al*. (2020), CC‐BY‐NC 3.0 (https://creativecommons.org/licenses/by‐nc/3.0/).

The issues that can be encountered in the large‐scale application of CLEAs are related to their physical properties, as the particles are not uniform (5–50 μm), are quite small (usually below 10 μm), and show low mechanical stability.^[7]^ To tackle these issues, the encapsulation of CLEAs in the polyvinyl alcohol hydrogel particles Lentikats® resulted in an improved operational stability of different biocatalysts.[^24^, ^25^] In another example, Mbanjwa *et al*. produced self‐immobilized enzyme microspheres (“spherezymes”) with a diameter of 50 μm and a size distribution within 3 %.^[26]^ Such preparations of lipase from *Pseudomonas* sp. were obtained by addition of a cross‐linker to a suspension of enzyme in a water‐in‐oil emulsion. Regulating the size of the microparticles offers several advantages; for instance, larger particles can be more easily recovered through filtration. Additionally, the ability to produce particles within a narrow diameter range is essential for ensuring consistent catalytic activity and allowing for standardized particle recovery methods. In un another work, De Martino *et al*. obtained a robust self‐immobilized biocatalyst system by encapsulating cross‐linked enzyme nano‐aggregates (CLEnA) into the cavity of bowl‐shaped polymer vesicles, named stomatocytes, made of poly(ethylene glycol)‐polystyrene (Figure 2).^[27]^ In the first step, the target enzyme was encapsulated into the stomatocytes, then the cross‐linker (glutaraldehyde or genipin) was added to obtain the CLEnA. Remarkably, a low amount of cross‐linking agents was necessary and that preserved enzyme activity. This nanoreactor was tested with *Candida antarctica* Lipase B and porcine liver esterase, as well as a mixture of glucose oxidase and horseradish peroxidase. The encapsulated CLEnA system demonstrated to be very robust, showed well‐defined nano‐sized particles, and could be applied in a nanoreactor flow system and reused for ten times without losing any activity.^[27]^

### 2.2 Heterogenous Biocatalysts by Enzyme Entrapment

Concerning entrapment‐based strategies, the support material is a solid or gel which is created or reformed in the presence of an enzyme.^[28]^ While the enzyme is not directly involved in the formation of this solid or gel, its presence can influence the process, acting as a template. As the matrix forms, the enzyme becomes encapsulated within it, typically through a combination of size effects and supramolecular forces. The resulting material is often a (hydro)gel, which consists of a solid cross‐linked network that contains a confined liquid. The enzyme usually maintains its native structure, since it undergoes minimal modification within the gel, and the liquid environment can be tailored to optimize the enzyme′s performance. However, challenges include the potential for enzyme leaching and mass transfer limitations, which can reduce the overall efficiency. Moreover, hydrogels frequently show a limited mechanical stability, which could hamper the development of scalable biocatalyzed batch and flow processes.^[29]^

In a recent work, after achieving limited success with covalent and ion‐affinity binding methods, Croci *et al*. explored the entrapment of amine dehydrogenase and formate dehydrogenase in agarose hydrogel.^[30]^ 3D printing was used to create custom molds for the agarose hydrogel, allowing the formation of a stable, continuous flow reactor which operated for 120 hours, achieving a 47 % analytical yield and a space‐time yield of 7.4 g^−1^ L^−1^ day in the reductive amination of benzaldehyde to benzylamine. However, a decrease in cascade reactor performance was observed after 48 h of operation which was ascribed to the leaching of the cofactor‐recycling enzyme formate dehydrogenase. The authors suggest that this issue could be faced by either improving the agarose concentration or fusing the two biocatalysts to form a chimera with an increased protein size.^[30]^

In another example, unspecific peroxygenase (UPO), an enzyme useful in biocatalytic stereoselective oxygenation reactions, was entrapped into the synthetic hydrogel polyethylene glycol diacrylate/[2‐(acryloxy)ethyl] trimethyl ammonia chloride (PEGDA/AETMA).^[31]^ In this case, no protein leaching was observed after repeated washing of enzyme‐loaded hydrogels. However, the activity yield of this heterogeneous biocatalyst was notably low (6.2 %), and its reusability fell significantly short of expectations. The authors attribute these limitations primarily to mass transfer effects that could be hopefully overcome by implementing the production of a highly porous support material.

Overall, these findings highlight the need for further research to address the observed limitations and pave the way for the practical application of 3D‐printed enzyme‐containing hydrogels in flow biocatalysis. As an alternative, self‐assembling all‐enzyme hydrogels have been recently reported[^32^, ^33^] and will be presented in the following (see paragraph 5.1.).

## 3 Recent Advancements in the Selection and Application of Suitable Materials for Enzyme Immobilization

Moving to support‐based enzyme immobilization, selecting the appropriate carrier is critical to enhance the properties of the immobilized enzyme. There is no universal rule for choosing the “perfect” carrier, as the selection process often involves a combination of trial and error. However, understanding the advantages and disadvantages of each type of material is essential for making an informed decision.

**Figure 3:**
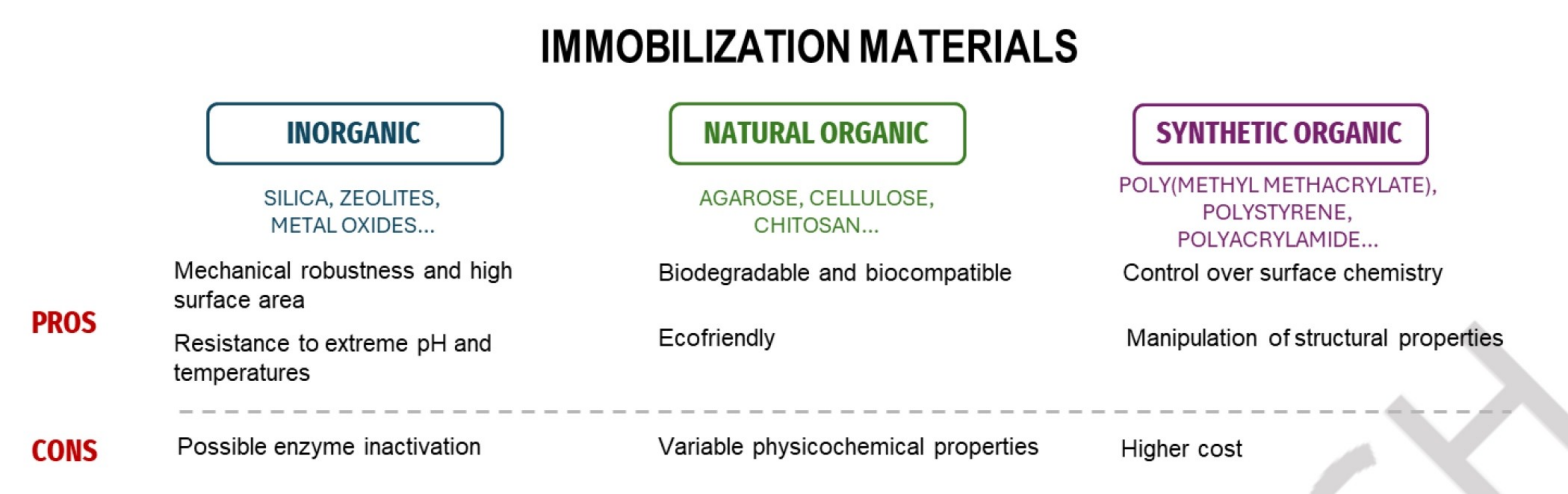
Overview of immobilization materials.

Carrier materials are generally classified into inorganic materials, natural and synthetic organic materials, each offering distinct advantages and limitations (Figure 3).^[5]^ Inorganic materials, such as silica, zeolites, and metal oxides, are prized for their mechanical robustness, high surface area, and resistance to extreme pH and temperature conditions, although they often face challenges regarding limited compatibility with the protein, which can lead to enzyme denaturation or reduced activity over time. For instance, surfaces like metal oxides or certain ceramics may need additional modification to prevent unfavorable enzyme‐material interactions.^[34]^ Natural organic materials, like agarose, cellulose, chitosan, and alginate, provide biodegradability and biocompatibility, making them suitable for green and sustainable applications, though their physicochemical properties can significantly vary depending on the source. Synthetic organic materials, including poly(methyl methacrylate) (PMMA), polystyrene, polyacrylamide, and polyurethane, allow for precise control over surface chemistry and structural properties, facilitating tailored enzyme immobilization and consistent performance, albeit at a higher cost. Critical properties of these carriers, such as porosity, hydrophilicity, and surface functionalization, significantly influence immobilization efficiency and the catalytic activity of the enzymes. High surface area and appropriate functional groups on the carrier surface are particularly beneficial for maximizing enzyme loading and maintaining high catalytic activity.^[35]^

In conclusion, despite their advantages, each type of organic carrier material presents specific challenges, such as the high cost and scalability issues of synthetic materials and the variability inherent in natural materials. Continuous research efforts are focused on developing innovative materials and techniques to overcome these limitations, aiming to enhance the efficacy and sustainability of enzyme immobilization in biocatalysis.^[7]^

Agarose and PMMA microbeads are among of the most widely used prefabricated material supports for purified enzyme immobilization. Agarose, composed of sugars, is more hydrophilic compared to PMMA, which is made by acrylic polymers. Its versatility is well‐documented, as demonstrated by numerous examples in the literature. For instance, Tentori *et al*. successfully immobilized the ene‐reductase OYE3 from the Old Yellow Enzyme family on glyoxyl‐agarose and employed it for the stereospecific reduction of activated prochiral C‐C double bonds.^[36]^ Similarly, the (*S*)‐selective amine transaminase from *Vibrio fluvialis* was successfully immobilized on this material and used under flow conditions for the synthesis of (*S*)‐1‐(5‐fluoropyrimidin‐2‐yl)‐ethanamine, a key intermediate of the JAK2 kinase inhibitor AZD1480.^[37]^

In a recent study, Benitez‐Mateos and Contente conducted a comparative analysis of agarose‐ and methacrylate‐based materials by investigating the retained activity, stability, and overall performance of eleven enzymes immobilized on these supports, demonstrating that the physicochemical properties of the matrix significantly influence enzyme performance.^[38]^ Their results indicate that biocatalysts immobilized on agarose retained higher activity, regardless of the enzyme type. Moreover, they observed that the hydrophobic nature of methacrylate‐based scaffolds affects the inertness of the carrier, leading to the stickiness of apolar compounds even under flow conditions.

Also in the case of inorganic materials, the hydrophilicity of the support can result helpful to avoid aspecific adsorption of substrates and products. This was the case for the development of an integrated chromatographic system to rapidly investigate in a continuous flow mode the biocatalytic properties of ω‐transaminases, where a wide‐pore epoxy silica monolithic support was selected to create an immobilized reactor (IMER) using the (*R*)‐selective ω‐transaminase ATA‐117 from *Arthrobacter* sp.^[39]^

However, there is no universal rule favouring one material over the other. In some cases, methacrylate‐based supports prove to be superior. For example, Patti *et al*. reported that the immobilization of the (*S*)‐selective transaminase from *Streptomyces* sp. BV333 resulted in an active immobilized derivative on the epoxy carrier Eupergit® C,^[40]^ while the same enzyme immobilized on glyoxyl‐agarose was not active in the biotransformation of interest.^[41]^

### 3.1 Innovative Perspectives in the Use of Natural Materials as Supports for Enzyme Immobilization

Recent advancements in materials for enzyme immobilization have focused on developing innovative carriers that enhance enzyme stability, activity, and reusability, all aspects of utmost importance in a sustainability view.

Enzyme immobilization supports based on natural sources can offer several advantages like good compatibility with enzymes, availability, and cost‐effectiveness. However, they can also come with certain disadvantages, such as a possible variability in composition, a limited physical and chemical stability, and the susceptibility to microbial contamination.

Lignin, an abundant and renewable biopolymer, has emerged as an attractive carrier due to its versatility and stability (both to high pHs and temperatures), as well as its eco‐friendliness, bioavailability, biodegradability, and non‐toxicity, making it a green alternative to fossil‐based materials.^[42]^

A recent study innovatively employed an aldehyde‐stabilization technique to extract and concurrently functionalize lignin, resulting in a series of lignin derivatives with a variety of reactive groups, including epoxy, amine, aldehyde, and metal chelates.^[43]^ This approach facilitated the immobilization of a diverse range of enzymes, such as carboxylases, dehydrogenases, and transaminases, through multiple binding chemistries, achieving immobilization efficiencies between 64 % and 100 %. To demonstrate the applicability of this method in a flow process, an ω‐transaminase was reversibly immobilized on polyethyleneimine‐lignin and utilized in a packed‐bed reactor. The immobilized enzyme exhibited remarkable stability in continuous‐flow deamination reactions, maintaining consistent conversion rates across 100 cycles. However, the activity recovery (that in various cases was lower than 10 %) needs to be improved and this challenge must be addressed to fully realize lignin′s potential in industrial applications.^[43]^

**Figure 4:**
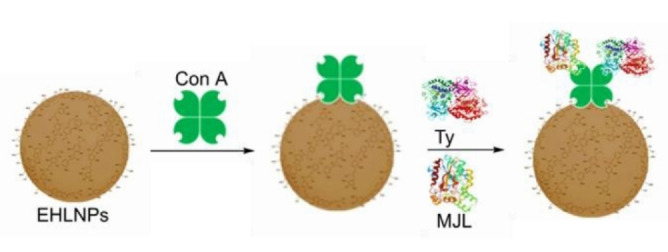
Preparation of co‐immobilized tyrosinase (Ty) and lipase (MJL) cascade using as support enzymatically hydrolyzed lignin nanoparticles (EHLNPs) and the lectin Concanavalin A (Con A) as spatial orienting agent. Reproduced with adaptation from,^[45]^ copyright Piccinino *et al*. (2023), CC‐BY 4.0 (https://creativecommons.org/licenses/by/4.0).

Concerning the immobilization of glycosylated enzymes, Tomaino *et al*. presented a detailed study on the development of a biocatalytic system using lignin nanoparticles to support a tyrosinase‐lipase enzymatic cascade to be employed in the synthesis of lipophilic hydroxytyrosol esters.^[44]^ The lectin Concanavalin A (Con A) functioned both as a molecular spacer between the support and the enzymes and ensured optimal orientation and spacing between the two enzymes (Figure 4). This precise arrangement facilitated the substrate‐tunneling effect, enhancing the overall catalytic efficiency. In a following work, the same binding approach was used to decorate hybrid melanin‐lignin nanoparticles with the tyrosinase‐lipase couple.^[45]^ The obtained heterogenous biocatalyst, thanks to the electrochemical properties of melanins, showed a significantly improved tyrosinase activity without the need of using additional reducing agents, thus emphasizing the potential to precisely tailor the support for optimized process performance.

**Figure 5:**
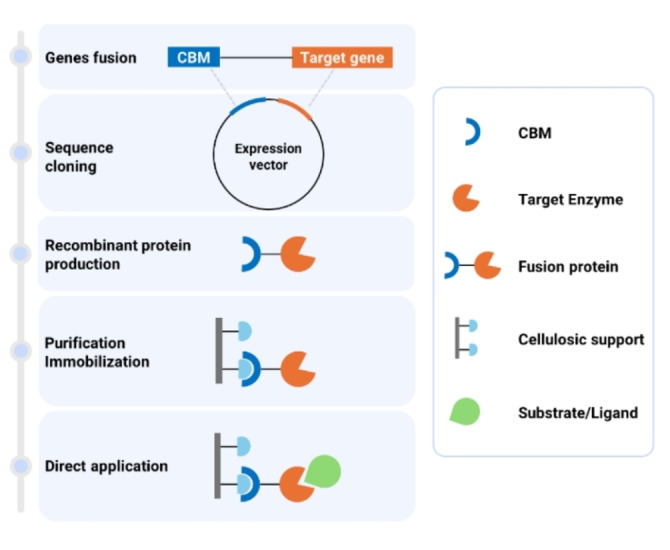
Diagram illustrating the recombinant Carbohydrate Binding Module (CBM)‐fusion technology, covering the entire process from cloning to application.

Moving to polysaccharides‐based supports, various studies have been carried out on the exploitation of Carbohydrate‐Binding Modules (CBMs), small non‐catalytic domains found in carbohydrate‐active enzymes and characterized by their strong affinity for specific carbohydrate structures. CBMs offer a promising approach for the one‐step purification and immobilization of enzymes since, by fusing CBMs with target enzymes, the enzymes can be directly bound to specific carbohydrate structures on various supports or surfaces, facilitating their immobilization (Figure 5).^[46]^

CBMs have shown great potential, especially when combined with cellulosic supports, that allow for high immobilization yields and preserve enzyme activity. Benito *et al*. investigated the use of CBM domains, in particular those that bind cellulose, for a one‐step purification and immobilization of enzymes.^[47]^ They fused either CBM3 domain from *Clostridium thermocellum* or CBM9 domain from *Thermotoga maritima* to an alcohol dehydrogenase from *Saccharomyces cerevisiae*, achieving immobilization yields over 98 % on low cost regenerated amorphous cellulose and retaining about more than 80 % of initial activity.

Another study evaluated the immobilization of the (*S*)‐selective transaminases from *Vibrio fluvialis* (VfTA) and *Chromobacterium violaceum* (CvTA) on Avicel® microcrystalline cellulose using a CBM from *Clostridium thermocellum* (CBDclos).^[48]^ Despite some activity loss and protein aggregation issues, a VfTA‐CBDclos fusion was successfully used in repetitive batch and continuous flow reactors. In particular, the repetitive batch reactors recycled the catalyst over seven consecutive one‐hour batches, achieving higher reaction yields and volumetric productivities, while the continuous flow reactor, fed with reagents for almost 10 hours, exhibited greater specific productivity and enzyme yields.

Recently, a CBM derived from the thermostable *Microbulbifer thermotelerans* agarase (MtCBM) was employed as a binding tag to enhance the retention of various biocatalysts in agarose hydrogels.^[49]^ By testing the MtCBM‐fused enzymes in flow experiments in a flat‐bed agarose reactor, an improvement in productivity was observed, likely attributable to the prevention of enzyme leaching from the support.

### 3.2 Biocatalysts Immobilization on Advanced Synthetic Materials

**Figure 6:**
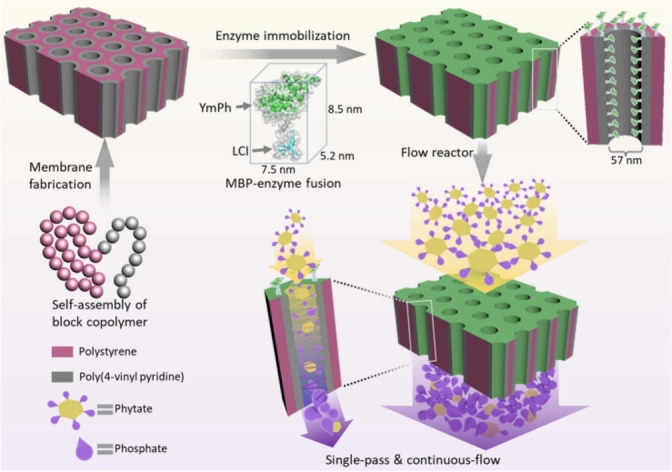
Scheme depicting the design of a flow reactor that utilizes isoporous block co‐polymer (BCP) membranes as carriers combined with a fusion protein of the phytase (YmPh) linked to a Material Binding Peptide (MBP). The system efficiently releases phosphate from phytate as the phytate feed solution flows through the nanochannels containing the immobilized enzyme in a continuous flow process. Reproduced from,^[50]^ copyright Zhang *et al*. (2024), CC‐BY 4.0 (https://creativecommons.org/licenses/by/4.0).

Block co‐polymers are innovative materials, designed with specific functional groups that enable tailored interactions with enzymes.^[50]^ These materials can facilitate enzyme immobilization while maintaining high activity and stability, moreover the flexibility in the design of block co‐polymers allows for the creation of supports that can be optimized for different enzymes and applications. Zhang *et al*. reported an example of generalizable, scalable, and robust flow reactor design concept. First, they developed and validated the fabrication of high‐performance, continuous‐flow enzyme‐functionalized nano‐and isoporous block co‐polymer (BCP) membrane reactor. Subsequently, they successfully immobilized a phytase from *Yersinia mollaretii* (YmPh), fused with a specific Material Binding Peptide,^[51]^ achieving high productivity (about 2000 mol of released phosphate per m^2^ membrane in 30 days) and a high space‐time yield (1.05×10⁵ g L^−1^ d^−1^) (Figure 6).^[50]^

Covalent Organic Frameworks (COFs), with their well‐defined porous structures and tunable chemistry, have also gained significant attention. COFs offer a high surface area and customizable pore environments, which can significantly enhance enzyme loading and stability. Their structural precision allows for the creation of highly efficient biocatalytic systems. In a recent paper, Paul *et al*. innovatively integrated enzyme purification and immobilization into a single, streamlined process through the development of a flow‐based technology utilizing a nickel‐infused covalent organic framework (Ni‐TpBpy COF).^[52]^ This method effectively allowed to purify various His‐tagged biocatalysts, such as β‐glucosidase, cellobiohydrolase, and endoglucanase. Notably, the enzymes maintained a catalytic activity comparable to that of free enzymes, demonstrated recyclability for over five cycles in small scale recycling assays, and exhibited long‐term stability at room temperature, surpassing the stability of their free forms in buffer. It might be foreseen that further studies will confirm in the future the practical applicability of these systems in synthetic processes.

### 3.3 Application of 3D‐Printed Materials for Enzyme Immobilization

3D printing technology allows for the creation of carriers with highly specific and customizable architectures, enabling precise control over enzyme loading and distribution. This technology also allows for the rapid prototyping and testing of new carrier designs, accelerating the development process.^[53]^

An extrusion‐based 3D printing technique was developed to embed thermostable enzymes in an agarose matrix, enabling multi‐step sequential biotransformations and chemo‐enzymatic conversions in flow reactors.[^54^, ^55^] While some enzyme leaching from the support was observed in the absence of specific binding tags, these studies lay the groundwork for integrating advanced manufacturing techniques into biocatalysis applications.

**Figure 7:**
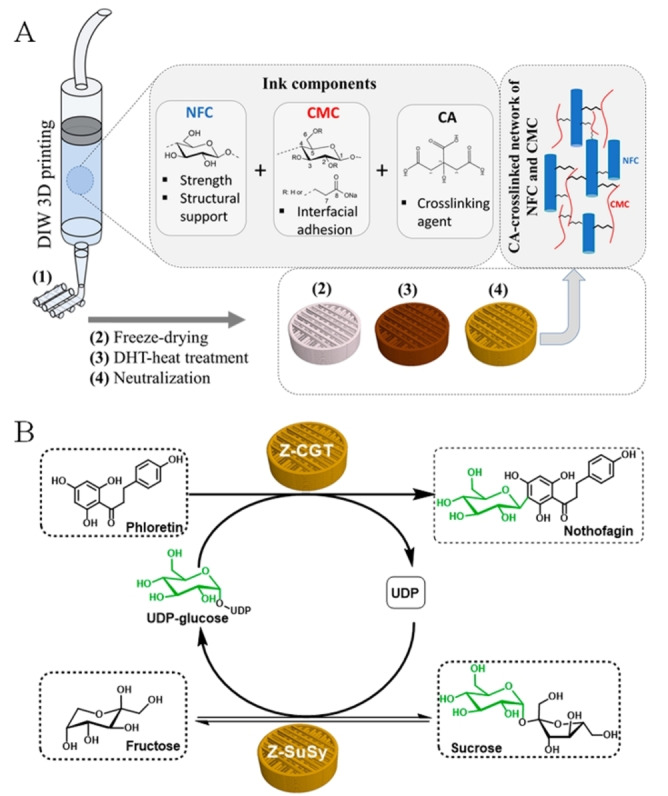
Co‐immobilization of *C*‐glucosyltransferase (CGT) and sucrose synthase (SuSy) on a 3D‐printed polysaccharide scaffold and their utilization in the cascade synthesis of the C‐glycoside nothofagin from phloretin. The two enzymes carried a cationic protein module (Z_basic2_) to allow the binding to the cross‐linked material containing nanofibrillated cellulose (NFC) and carboxylmethyl cellulose (CMC). Reproduced from,^[56]^ copyright Lackner *et al*. (2022), CC‐BY 4.0 (https://creativecommons.org/licenses/by/4.0).

In a recent work, Lackner *et al*. developed and evaluated 3D‐printed nanocellulose‐based scaffolds for the immobilization of *C*‐glucosyltransferase (CGT) from rice (*Oryza sativa*) and sucrose synthase (SuSy) from soybean (*Glycine max*). The obtained co‐immobilized enzyme preparation was then used in the cascade bioconversion of phloretin into the C‐glycoside nothofagin (Figure 7).^[56]^

The study highlights the versatility of the scaffolds, capable of supporting co‐immobilization of multiple enzymes. Additionally, the tunable properties of the scaffolds, such as pore size and charge, are emphasized as critical for optimizing enzyme performance. Although some challenges arise from the limited post‐immobilization activity recovery and reusability (recycling was possible for up to five consecutive reactions, but with gradually decreased conversions), this work showcases a promising approach for enzyme immobilization in biocatalysis.

Overall, these recent advancements in material science are driving significant improvements in enzyme immobilization techniques, enhancing the efficiency and applicability of biocatalysis across various industries.

## 4 Smart Utilization of Non‐covalent Immobilization Strategies

Non‐covalent attachment of the enzyme to the matrix surface can occur through short‐term, low‐energy interactions such as van der Waals forces, dipole interactions, and London dispersion forces, or through long‐term, high‐energy interactions like ionic bonds.^[57]^

Unlike covalent methods that might alter the enzyme′s active site or overall conformation, non‐covalent interactions, such as adsorption, ionic binding, and affinity interactions, typically maintain the enzyme′s native structure and function. Various examples can be easily found where the enzyme′s activity after immobilization has either remained unchanged or has resulted in increased stability at extreme pH and temperature. For example, studies have shown that lipases immobilized through adsorption onto hydrophobic supports retain nearly 100 % of their initial activity.[^58^, ^59^] Similarly, β‐galactosidase from *Aspergillus oryzae* immobilized on ion‐exchange resins has demonstrated higher activity at pH 5 and 9 compared to its free form.^[60]^

Another significant advantage of non‐covalent immobilization for biocatalytic applications is the ability to either bypass or combine enzyme purification, making it an attractive option for large‐scale production and diverse biotechnological implementations.^[10]^ Since this not only simplifies the overall process but also reduces the time and cost associated with enzyme exploitation, this interesting aspect will be the focus of the following selected examples on non‐covalent immobilization aimed to develop more performing biocatalysts with longer operational life‐span and improved cost‐effectiveness.

### 4.1 Optimized Protocols for Enzyme Immobilization by Adsorption on Charged Supports

**Figure 8:**
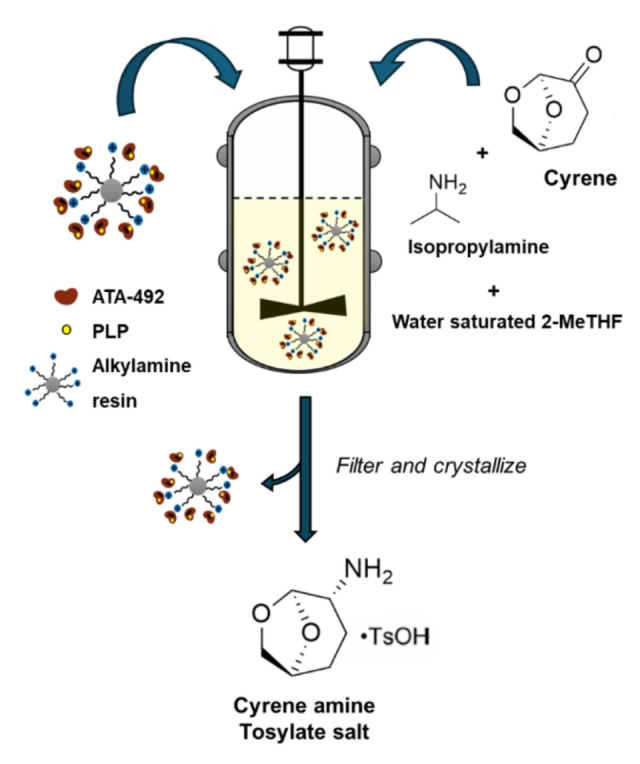
Transamination of cyrene catalyzed by the amine transaminase ATA‐492, immobilized by adsorption on an alkylamine functionalized methacrylate‐based resin, in the presence of isopropylamine as amine donor and water‐saturated 2‐methyl tetrahydrofuran (2‐Me THF) as reaction medium. The cyrene amine product was recovered by filtration of the immobilized biocatalyst and precipitation with *para*‐toluensulfonic acid.^[61]^

Recently, Merck scientists have conducted an extensive evaluation of various commercially available resins for the immobilization of the transaminase ATA‐492 from lyophilized *E. coli* cell‐free extract.^[61]^ Their molecule of interest, obtained from the biocatalytic transamination of the green solvent cyrene, is the chiral derivative cyrene amine (Figure 8), a key intermediate in the synthesis of the drug Nemtabrutinib. The immobilization of the enzyme allowed the use of 2‐methyl tetrahydrofuran (2‐MeTHF) as reaction medium, simplifying the isolation process of the highly water‐soluble product and avoiding enzyme leaching. Resins with hydrophilic methacrylate‐based matrices and alkylamine linkers (ECR8309, ECR8409, ECR8415) showed superior performance, yielding high amine product compared to the unfunctionalized resin (conversion: 86 % and 60.6 %, respectively). Moreover, the enzyme was completely immobilized following a 5‐hour incubation. The improved performance was attributed to selective enzyme binding at pH 6.8 due to opposite charges between the enzyme, showing a calculated isoelectric point (pI) of 4.9, and amino‐derivatized resins, resulting in an enrichment of the enzyme over host cell proteins by about 50 %.

A combination of enzyme engineering and process optimization allowed these researchers to set up the multigram‐scale synthesis of cyrene amine in a rotating bed reactor. By a simple crystallization, the product was isolated in good yield and high diastereomeric ratio (*dr*) (51 : 1).^[61]^ Noteworthily, in a following study, the Merck research group successfully translated the batch process into a dynamic flow system, producing cyrene amine continuously from 26 kilograms of cyrene and with an even improved *dr* (200 : 1).^[62]^

**Figure 9:**
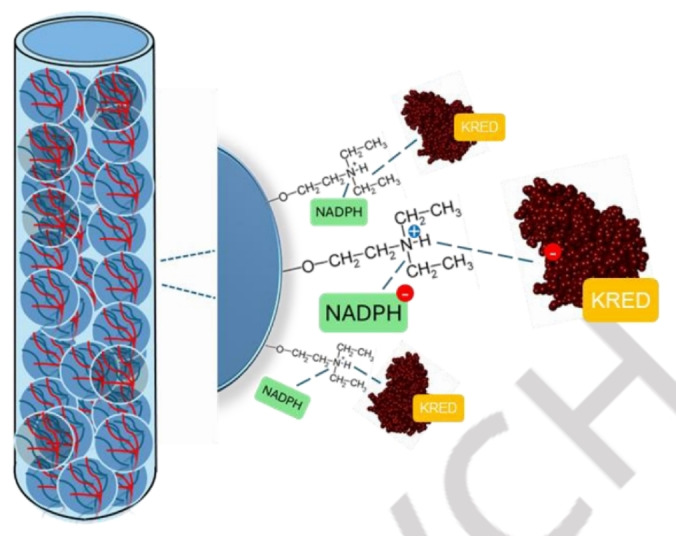
A self‐sufficient heterogeneous biocatalyst obtained by co‐immobilization of the ketoreductase P1‐A04 (KRED) and its cofactor NADPH on diethyl aminoethyl (DEAE) functionalized agarose beads.^[63]^

The study of Benitez‐Mateos *et al*. shows another example of how a diethylaminoethyl (DEAE)‐based ion exchange resin can be exploited to simultaneously purify and immobilize a protein from a cell‐free extract.^[63]^ Ketoreductase P1‐A04 (KRED) and its cofactor NADPH were co‐immobilized on DEAE‐activated agarose beads, enabling self‐regeneration of NADPH through isopropyl alcohol oxidation catalyzed by the same ketoreductase (Figure 9).

Remarkably, while in most cases the KRED under study lost its activity after covalent immobilization on other supports,^[63]^ the developed self‐sufficient heterogeneous biocatalyst allowed the asymmetric reduction of a series of ketones and aldehydes to the corresponding secondary and primary alcohols with excellent yields and without exogenous addition of the redox cofactor. Moreover, the excellent reusability of the immobilized enzyme on batch led the continuous asymmetric reduction of selected aryl ketones in a packed‐bed flow reactor. The reactor ran for 120 hours, achieving ~80 % yield without enzyme inactivation or cofactor leaching.^[63]^

### 4.2 His‐Tag‐Based Enzyme Immobilization Strategies

Several examples of non‐covalent enzyme immobilization are related to the use of pseudo‐affinity strategies, such as that derived from immobilized metal affinity chromatography (IMAC), a technique first described by Porath *et al*. in 1975^[64]^ and since then widely used to purify numerous proteins and peptides, primarily utilizing transition metal ions such as Ni^2+^, Zn^2+^, Cu^2+^, and Co^2+^. This non‐covalent immobilization technique is highly selective towards His‐tagged fused protein^[65]^ which, if necessary, can be detached by simply using high concentrations of imidazole.

Conventional protein immobilization methods generally lack precise control over enzyme orientation on solid carriers, often leading to unfavorable conformational changes that promote enzyme deactivation. Instead, His‐tag immobilization frequently prevents interference with the active site as recently described by Basso *et al*.^[66]^ In this work, a NADPH‐dependent ketoreductase was co‐immobilized on a methacrylate iminodiacetic (IDA) resin (Chromalite MIDA), loaded with either Co^2+^, Ni^2+^, Cu^2+^, or Fe^2+^ metal ions, with a glucose dehydrogenase for cofactor recycling. The obtained biocatalysts showed up to three times higher activity compared to other immobilization methods, such as covalent (epoxy), ion exchange (amino resin), and adsorption on styrene or divinylbenzene/methacrylate resins.

**Figure 10:**
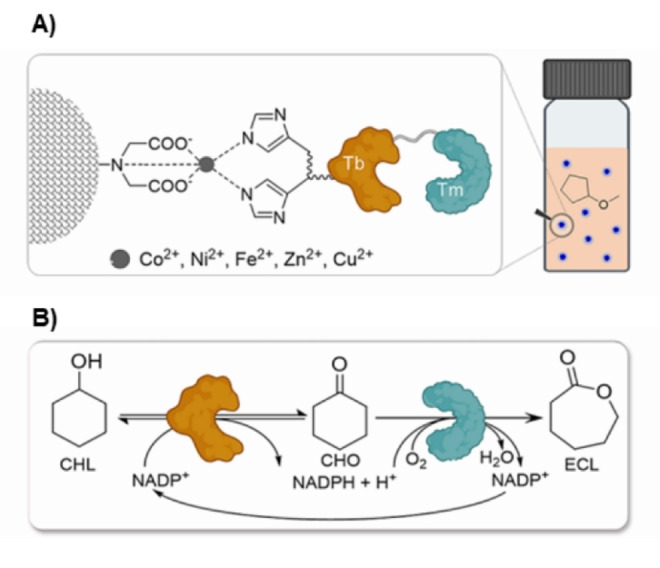
Immobilization of fused alcohol dehydrogenase (Tb) and cyclohexanone monooxygenase (Tm) on a methacrylate iminodiacetic resin (Chromalite MIDA) loaded with various metal ions (A) and its application in the cascade oxidation of cyclohexanol (CHL) to ε‐caprolactone (ECL) (B), Reprinted and adapted with permission from.^[67]^ Copyright 2024 American Chemical Society.

The same MIDA resin was subsequently applied for the immobilization of the fusion enzyme consisting of cyclohexanone monooxygenases and alcohol dehydrogenases (Figure 10).^[67]^ After optimization of the immobilization process by Design of Experiments (DoE) to maximize both activity and immobilization yields, the catalytic efficiency of the immobilized enzyme fusion was tested in a linear cascade reaction to synthesize the polymer precursor ε‐caprolactone from cyclohexanol. The immobilized fused biocatalysts demonstrated enhanced productivity of ε‐caprolactone in 99.5 % (*v*/*v*) cyclopentyl methyl ether compared to a buffer system. Moreover, it exhibited higher operational stability than the free enzyme, being reused for up to seven cycles, and a good potential for process scale‐up, as demonstrated by preliminary investigations in a 125 mL rotating bed reactor.^[67]^

**Figure 11:**
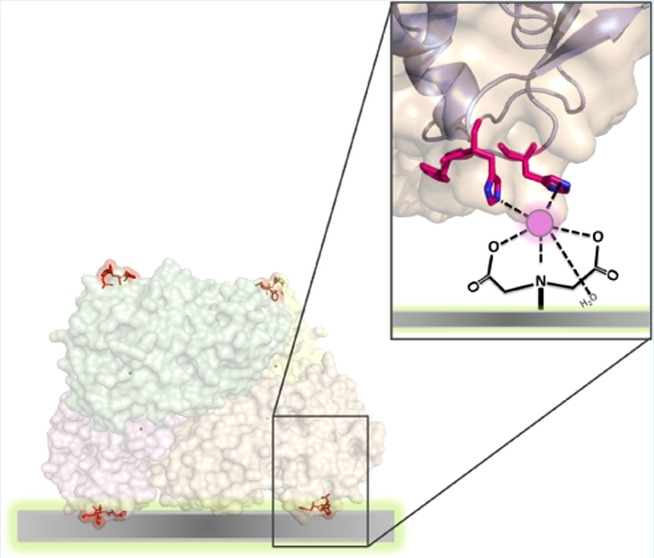
Exploitation of histidine clusters on specific enzyme flexible regions for the obtainment of region‐oriented heterogenous biocatalysts. Reproduced from,^[68]^ copyright Zeballos *et al*. (2024), CC‐BY‐NC‐ND 4.0 (https://creativecommons.org/licenses/by/4.0)

Undoubtedly, the only way an enzyme can fully keep its activity unaltered after immobilization is by displaying a correct orientation and, even if infrequent, there are some cases where enzymes attached by the His‐tag at the N‐ or C‐terminal could still experience a loss of activity. In a recent study by the López‐Gallego′s group, the significance of orientation was examined by modifying the surfaces of two model dehydrogenases, introducing histidine clusters into flexible regions that do not participate in the catalysis (Figure 11).^[68]^ Based on structural evaluation derived from thermodynamics simulations, five protein loops were selected for the introduction of histidine clusters composed of five histidine residues. For certain His‐clustered variants, specific orientations resulted in greater (thermo)stability and better recycling performance of the immobilized enzymes compared to their His‐tagged counterparts on identical carriers (agarose microbeads activated with Co^2+^).

Alternative plastic‐free carriers, namely EziG^TM^ (EnginZyme AB, Sweden), that exploit the interactions between His‐tagged proteins and metal ions, were developed.^[69]^ These materials consist of controlled pore glass coated with an organic polymer and chelated Fe(III) ions, which enable the selective binding of His‐tagged proteins directly from crude cell lysate. Although it is worth mentioning that in some cases protein leaching was observed during flow processes,[^30^, ^70^] thanks to efficient mass transfer through interconnected pores and selective, non‐destructive His‐tag binding, a high enzyme mass loading can be achieved without the loss of activity typically caused by diffusion limitations and deactivation.^[71]^

EziG™ are available in three versions with different surface hydrophobicity: Opal (hydrophilic), Coral (hydrophobic), and Amber (semi‐hydrophilic).^[71]^ Generally, the immobilization of a specific enzyme is tested on all three materials because the immobilization yield and residue activity can vary between them.[^36^, ^72^, ^73^] Moreover, EziG^TM^ carriers perform well in flow setups, as well as in the presence of organic solvents. For example, a multienzymatic system has been recently developed for the continuous synthesis of chiral model compound (*R*)‐*N*‐(1‐phenoxypropan‐2‐yl)acetamide by immobilizing on EziG^TM^ Amber both the (*R*)‐selective transaminase from *Arthrobacter* and the lipase from *Candida antarctica*.^[74]^ Satisfactory process performance were achieved by separately packing the two immobilized enzymes into two different columns being part of the same flow system and using ethyl acetate as the reaction medium with a minimal water content not interfering with the lipase‐catalyzed acylation.

The same research group has lately reported an innovative biocatalytic system combining an ene‐reductase (ERED) from *Zymomonas mobilis* and an alcohol dehydrogenase from *Thermoanaerobacter ethanolicus* immobilized on EziG^TM^ supports, the Coral carrier being the most suitable in this case.^[75]^ Using the reduction of cyclohex‐2‐enone to cyclohexanol as model reaction, this system was tested in bulk organic solvents, an environment that has not been widely explored with EREDs due to challenges with cofactor recycling and enzyme activity. The maintenance of enzyme activity was successfully achieved by controlling of water content and optimizing specific post‐immobilisation treatments. However, some challenges were observed concerning the need of cofactor supplementation and limited biocatalysts stability during recycling, thus suggesting that further improvements are necessary to make the process applicable.

## 5 Using Genetic Fusion Technologies for Enzyme Immobilization

Protein tags are peptide sequences, either short or long, that are genetically fused to recombinant proteins for a variety of purposes, such as affinity purification, protein localization, and general labeling techniques.^[76]^

### 5.1 Application of Catcher/Tag Systems in Enzyme Immobilization

**Figure 12:**
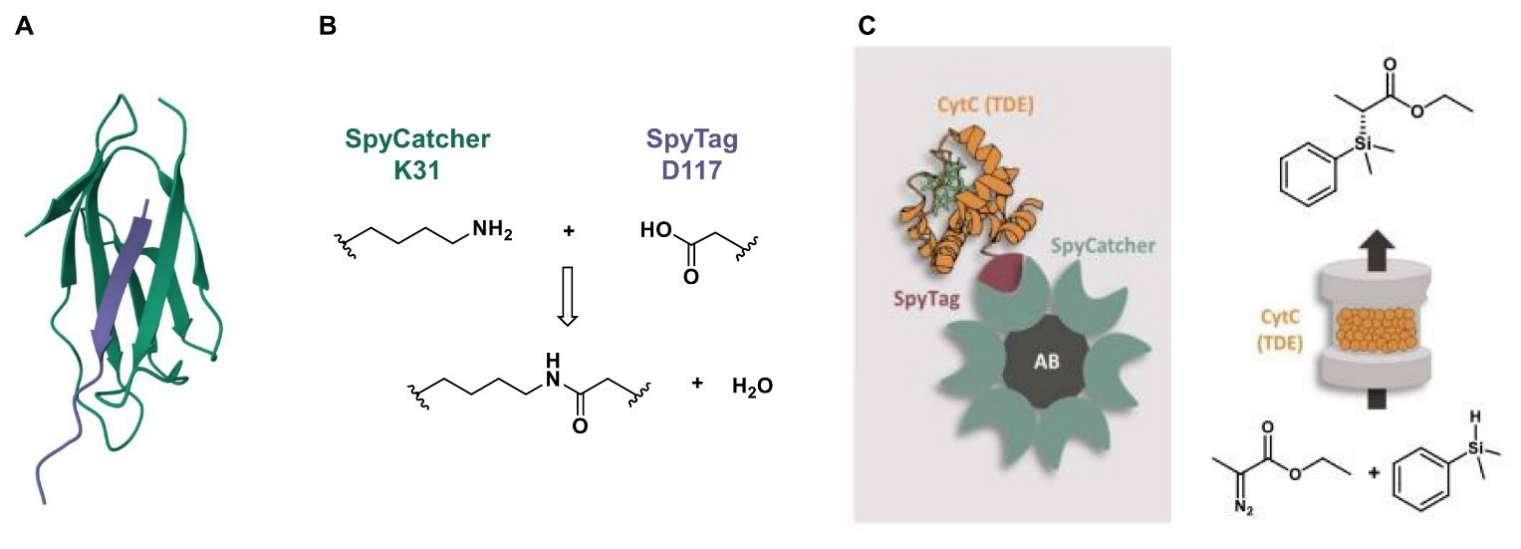
A) Crystal structure of the SpyTag/SpyCatcher complex (purple and green, respectively) (PDB: 4MLI); B) isopeptide bond formation between a specific lysine in SpyCatcher (K31) and an aspartic acid in SpyTag (D117); C) Immobilization of a SpyTagged cytochrome c variant [CytC(TDE)] on SpyCatcher‐functionalized agarose beads (AB) (left side) and application of the immobilized biocatalyst in the flow synthesis of organosilicon (right side, up) from ethyl 2‐diazopropanoate and phenyl dimethylsilane (right side, bottom), reproduced and adapted from,^[86]^ copyright Gallus *et al*. (2023), CC‐BY 4.0 (https://creativecommons.org/licenses/by/4.0).

The Catcher/Tag systems are protein engineering tools used for creating covalent bonds, typically isopeptide bonds, between proteins or protein/peptide pairs. Among them, the first and best studied system is that involving the 13 amino acids (aa)‐long peptide SpyTag and the 116 aa‐long protein SpyCatcher (Figure 12A), both derived by the splitting of the CnaB2 domain of fibronectin‐binding protein FbaB from *Streptococcus pyogenes*.[^77^, ^78^, ^79^] When SpyTag and SpyCatcher are mixed, they spontaneously form an irreversible isopeptide bond between a lysine (K31) in SpyCatcher and an aspartic acid (D117) in SpyTag (Figure 12B).

Drawing inspiration from the SpyCatcher/SpyTag system, various protein/peptide pairs that can spontaneously form isopeptide bonds were developed. These pairs were created through several methods, e. g., by splitting different CnaB domains, altering the split site, or by directed evolution of previously described Catcher/Tag couples.^[79]^ Thanks to the high efficiency, stability and selectivity of isopeptide bond formation, the currently available Catcher/Tag systems provide reliable methods for creating stable protein conjugates and assemblies. Therefore, they have been widely reported in the recent literature for applications in different fields, such as vaccine development,^[80]^ cyclization‐induced enzyme stabilization,[^81^, ^82^] cell imaging and labeling.[^77^, ^78^] Additionally, in the so‐called Spy&Go system, a non‐reactive SpyCatcher variant (SpyDock) was developed to be used in the purification of SpyTagged proteins.^[83]^

The Catcher/Tag systems have found interesting applications for the immobilization of enzymes to different types of surfaces. For example, SpyCatcher/SpyTag systems enabled the directional immobilization of enzymes, including the industrial enzyme glutaryl‐7‐aminocephalosporanic acid acylase (GA), on commercially available epoxy and agarose resins.^[84]^ In the case of epoxy resins, following the immobilization of SpyCatcher on the beads surface, very high immobilization efficiency and activity recovery of the Spy‐Tagged GA were achieved (>90 % and >85 %, respectively), even when starting from crude cell lysate. In a following study, SpyCatcher was immobilized on glyoxyl agarose, thus allowing the selective covalent immobilization of SpyTag‐fused enzymes from crude extracts, including a L‐phenylserine aldolase and a leucine dehydrogenase. An engineered version of SpyCatcher (mSC) was developed to enhance immobilization efficiency, resulting in improved binding capacity and thermal stability of the immobilized proteins. The immobilized biocatalysts showed an operational stability up to 8 reuse cycles.^[85]^

Additionally, some recent reports from the Niemeyer′s group concern the application of SpyCatcher/SpyTag systems in flow biocatalysis.[^32^, ^33^, ^86^, ^87^, ^88^] For example, an engineered variant of cytochrome c from *Rhodothermus marinus* [CytC(TDE)] was fused with a SpyTag, immobilized on SpyCatcher‐functionalized agarose beads, then used in a flow process to produce an organosilicon (Figure 12C).^[86]^ This immobilization did not significantly affect the enzyme′s catalytic activity, maintaining 60 % activity post‐immobilization. Moreover, the immobilized enzyme demonstrated up to a six‐fold increase in turnover number over a period of ten days compared to the free enzyme. In other cases, the SpyCatcher/SpyTag systems were applied to couples of enzymes showing a homotetrameric quaternary structure, i. e., *Lactobacillus brevis* alcohol dehydrogenase (ADH) and *Bacillus subtilis* glucose dehydrogenase (GDH).[^32^, ^33^, ^89^] Instead of using a solid support for enzyme immobilization, all‐enzyme hydrogels are formed by self‐assembling of SpyCatcher‐fused ADH with SpyTagged GDH. Recently, the application of such hydrogels in flow reduction reactions was studied either in *in vivo‐*based *E. coli* systems,^[32]^ or after their transformation in monodispersed dried foams.^[33]^ In both cases, the resulting enzymatic networks showed biocatalytic activity, although further investigations are presumably necessary to demonstrate their practical applicability in preparative biotransformations. An interesting application of all‐enzyme hydrogels in inline NMR monitoring of biocatalytic reactions has been also recently suggested.^[90]^

As an alternative to the pairing of Catcher‐modified supports with Tagged enzymes, the development of a modular magnetic nanoparticles (MNPs) platform functionalized with SpyTag, which allows for the immobilization of SpyCatcher‐fused proteins, has been recently investigated as well.^[91]^ This preliminary work, carried out with the fluorescent model proteins EGFP and RFP, could pave the way for further studies with synthetically useful enzymes.

### 5.2 Covalent Immobilization of Enzymes Using Self‐labeling Protein Tags

Self‐labeling protein tags are small proteins, typically less than 40 kDa, engineered for covalent attachment to a small‐molecule probe that is functionalized with a bioorthogonal linker.^[92]^ While these tools have been mainly developed to label, purify, and study proteins within cells or *in vitro*, they find interesting application in enzyme immobilization as well.

Among others, SNAP‐tag is a 20 kDa self‐labeling protein tag derived from the human DNA repair enzyme O^6^‐alkylguanine‐DNA alkyltransferase (hAGT).^[93]^ hAGT removes alkyl groups from the O^6^‐position on guanine bases through a unique single‐step mechanism known as “suicide enzyme” activity. In this process, the alkyl group from the damaged guanine is irreversibly transferred to a catalytic cysteine residue within the enzyme′s active site, effectively repairing the base, although permanently inactivating the enzyme at the same time.

**Figure 13:**
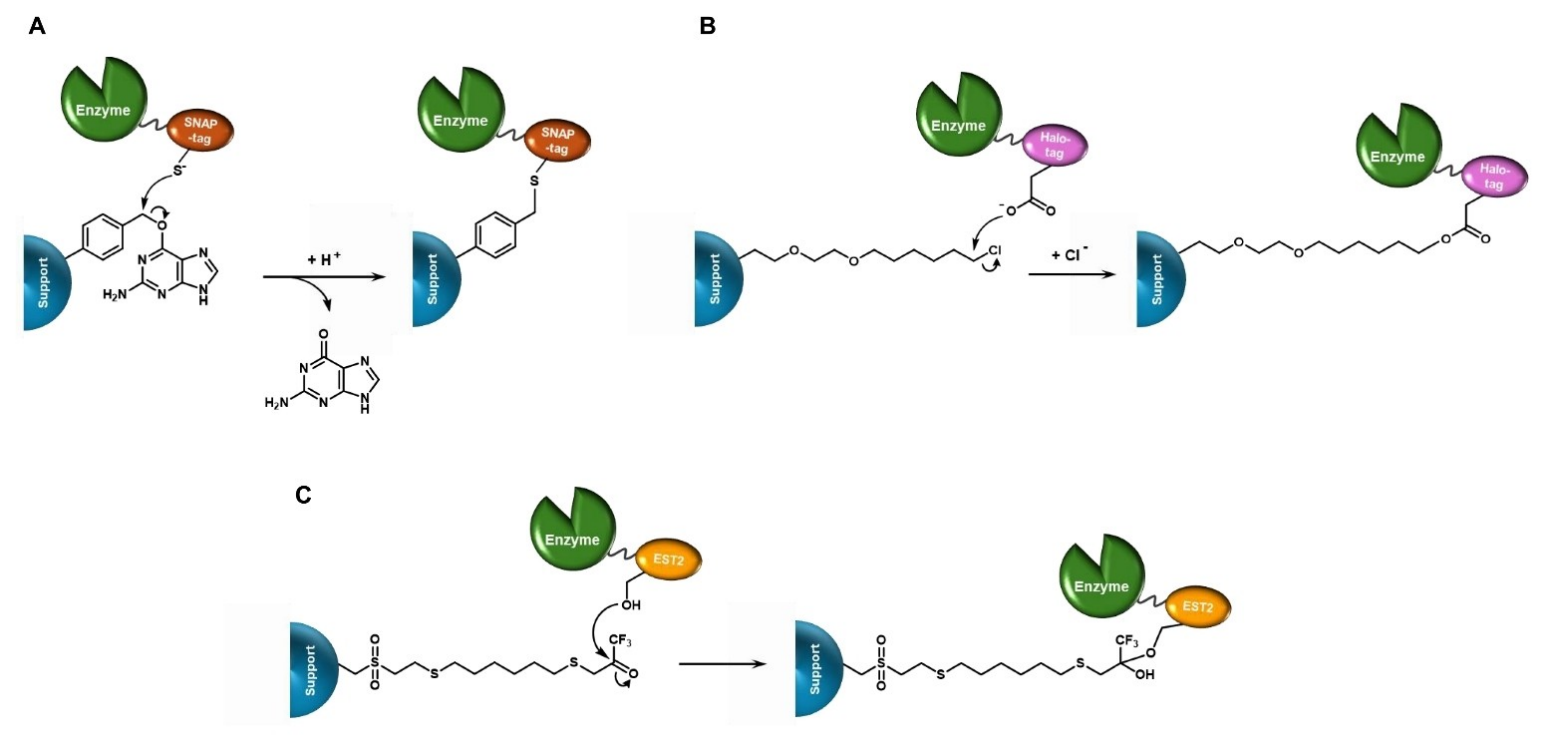
Mechanism of covalent immobilization of enzymes using genetically encoded SNAP‐tag (A), Halo‐tag (B), and EST2‐based tag (C).

In a similar way, when a support is functionalized with O^6^‐benzylguanine (BG) derivatives, a SNAP‐tagged target enzyme can be covalently linked to it following the same reaction mechanism (Figure 13A). An engineered variant of SNAP‐tag, named CLIP‐tag, that specifically recognizes O^2^‐benzylcytosine as substrate, was subsequently developed,^[94]^ thus, in principle, allowing the possibility of positional control in the binding of tagged proteins on the same support.

The SNAP‐tag technology was used by the patenting company New England Biolabs to achieve the covalent immobilization of DNA replication and modifying enzymes on magnetic beads.[^95^, ^96^] To improve enzyme activity, the beads’ surface was modified with polyethylene glycol (PEG). PEG served as both a surface coating and a spacer separating the enzyme from the bead surface. This modification contributed to a more hydrophilic environment and minimized nonproductive interactions between the enzyme and the solid support. These immobilized enzymes have been practically applied in next‐generation sequencing (NGS) library preparation, particularly enhancing read coverage in AT‐rich regions.^[96]^

Recently, new thermostable SNAP‐ and CLIP‐tags derived from hyperthermophilic organisms, such as *Saccharolobus solfataricus*, *Thermotoga neapolitana* and *Pyrococcus furiosus*, were discovered and characterized.[^97^, ^98^] These protein tags are intended for use in biotechnological applications, e. g., *in vivo* studies, that require extreme conditions, such as high temperatures. In agreement with their origin, they showed a superior catalytic activity and thermostability of compared to the corresponding mesophilic ones, thus paving the way to their application in enzyme immobilization as well.

Another tag that has garnered significant interest for its application in enzyme immobilization is Halo‐tag, a protein tag developed by Promega in 2008 and derived from a bacterial promiscuous haloalkane dehalogenase (DhaA) found in *Rhodococcus*.^[99]^ The wild‐type DhaA protein catalyzes the hydrolysis of a chloroalkane substrate through an S_N_2 reaction mechanism, which involves a Glu‐His‐Asp catalytic triad. Initially, the active‐site aspartate displaces the substrate′s halogen, forming an ester intermediate. This intermediate is then hydrolyzed via nucleophilic acyl substitution, with a water molecule activated by the active‐site histidine acting as the nucleophile. To create Halo‐tag, an engineered DhaA was designed by substituting the histidine residue with a catalytically inactive phenylalanine through mutagenesis, resulting in the DhaA(H272F) mutant that irreversibly captures the covalent ester intermediate.^[99]^ Following the same mechanism, a Halo‐tagged enzyme can be covalently linked to a solid support functionalized with a suitable chloroalkane derivative (Figure 13B).

Using the commercially available HaloLink^TM^ resin (Promega), the utility of this fusion tag in biocatalysis was first showcased using the thiamine diphosphate (ThDP)‐dependent enzyme benzaldehyde lyase from *Pseudomonas fluorescens* as a case study.^[100]^ Subsequently, the effectiveness of the Halo‐tag system for efficient enzyme immobilization and continuous biocatalytic production processes was shown by immobilizing a cascade of fusion enzymes directly from crude cell extracts in a packed‐bed reactor.^[101]^ Specifically, the study concerned a two‐step continuous enzymatic cascade for producing a chiral vicinal diol. The first step involved the decarboxylation of benzoylformate by a variant of *Pseudomonas putida* benzoylformate decarboxylase to produce (*S*)‐2‐hydroxy‐1‐phenylpropane‐1‐one, while, in the second step, this intermediate product was reduced by the *Lactobacillus brevis* alcohol dehydrogenase to the final product, (1*S*,2*S*)‐1‐phenylpropane‐1,2‐diol ((*S*,*S*)‐PPD). For both enzymes, the recovery yields were in a comparable range with other covalent immobilization methods. The immobilization could be directly performed in flow, then the two bioreactors were properly combined to carry out the enzymatic two‐step cascade. The optimized system achieved the continuous production of (*S*,*S*)‐PPD with high conversion rates and stereoselectivities (up to 99 % and 96 %, respectively), and significant space–time yields (up to 1850 g L^−1^ d^−1^).^[101]^

Recently, the Halo‐tag system was used for the covalent immobilization of Fe(II)/α‐ketoglutarate‐dependent dioxygenases (KDOs), an enzyme family useful for selective C‐H oxidation reactions, but showing challenges concerning their production in recombinant form and instability in purified form.^[102]^ In this study, the immobilization of three KDOs (CaKDO, CpKDO, FjKDO), which stereoselectively hydroxylate the L‐lysine side chain, was investigated by using different methods. The Halo‐tag‐based immobilization showed the best results in terms of residual activity and stability and the enzyme preparations were successfully applied on a preparative lab‐scale. Additionally, a Halo‐tagged immobilized lysine decarboxylase from *Selenomonas ruminantium* was utilized to convert the (3*S*)‐hydroxy‐L‐lysine produced by CaKDO into (2*S*)‐hydroxy‐cadaverine in a 15 mL consecutive batch reaction and without intermediate product purification.^[102]^

In an interesting comparative study, various immobilization techniques (Halo‐tag‐based covalent binding, His‐tag‐based non covalent binding, epoxy‐based covalent binding and Strep‐tag‐based affinity binding) were evaluated for the enzyme 2‐deoxy‐D‐ribose‐5‐phosphate aldolase (DERA).^[103]^ Since the performance of His‐tagged and Halo‐tagged immobilized DERA resulted comparable, Halo‐tag‐based immobilization was suggested by the authors as a reliable option if stability issues, such as biocatalyst leaching, arise.

Finally, it is worth mentioning the recent development of a novel method for the immobilization and display of proteins on polyacrylamide hydrogel beads (PHD beads) prepared by using a microfluidic droplet generator.^[104]^ The PHD beads were decorated by methacrylate‐PEG‐benzylguanine and methacrylate‐PEG‐chloroalkane anchors, which allowed for the covalent binding of SNAP‐ and Halo‐tagged fusion proteins, respectively. The binding occurs across the bead surface and within its volume, achieving a high density of protein display, approximately 1.5×10^9^ protein molecules per 20 μm bead, surpassing the capacity of traditional surface‐modified beads. Although the main applications of this technology are so far mostly related to bioassays, it might be foreseen that this precise and highly controlled enzyme immobilization approach will be further investigated in the future for synthetically useful biotransformations.

A third interesting example of genetically fused tag is the one related to the 34 kDa esterase EST2 from *Alicyclobacillus acidocaldarius*.[^105^, ^106^] EST2 is a thermostable and monomeric carboxylesterase, that can be easily expressed in soluble and active form in *E. coli* and shows a typical Ser‐His‐Asp catalytic triad in its active site. The immobilization technique that makes use of EST2 employs a suicide inhibitor, trifluoroketone (TFK), which reacts with the active site serine residue of the esterase to form a covalently bound “transition state mimic” (Figure 13C).[^107^, ^108^, ^109^] The formed bond anchors the esterase—and, by extension, the entire biocatalyst complex—to the support, ensuring that the enzyme remains stably immobilized and active throughout the reaction process.

EST2 has been recently applied to the development of modular biocatalysts designed for continuous‐flow biocatalysis, which retains and regenerates their cofactors.^[110]^ The process targeted was a three‐step enzymatic cascade designed to synthesize an advanced intermediate of the antidiabetic drug D‐fagomine from glycerol and 3‐aminopropanal. To achieve this, a modular multi‐enzyme fusion protein was engineered for each biocatalytic step. These proteins consisted of three key components: a catalytic module (responsible for the chemical reaction), a cofactor‐recycling module (responsible for regenerating the cofactor), and an immobilization module (responsible for anchoring the enzyme to a solid support), represented by EST2. Importantly, EST2 exhibited minimal impact on the kinetic behavior and thermostability of the other fused enzymes, both in solution and after immobilization. The flow bioreactors were prepared using as the support vinyl sulfone‐decorated agarose beads linked to a TFK derivative. The esterase module demonstrated exceptional efficiency in immobilization, achieving yields between 86 % and 98 %, depending on the specific biocatalyst. This high efficiency suggests that most of the esterase used in the reaction successfully formed the desired covalent bonds with the surface, thereby maintaining the functionality of the immobilized enzyme.

## 6 Summary and Outlook

This review provides an overview of the latest advances in enzyme immobilization techniques, including carrier‐free methods, entrapment strategies, and support‐based approaches, which are critical for the development of high‐performing and sustainable biocatalyzed batch and flow processes of interest for the fine chemicals and pharmaceutical industry.

In this context, we have examined the selection of appropriate materials for enzyme immobilization, emphasizing the benefits and challenges of using inorganic, natural, and synthetic organic carriers. Additionally, emerging opportunities from innovative binding strategies, such as smart non‐covalent adsorption approaches and genetic fusion technologies, aimed at the development of heterogeneous biocatalysts with improved activity and stability, have been explored. The review highlights the importance of continued research to overcome current limitations and optimize immobilization techniques for industrial applications.

At this regard, the choice of an immobilization method must be carefully tailored to the specific enzyme and overall process requirements. Selecting the appropriate approach can offer substantial advantages, including enhanced enzyme activity, improved operational stability, reusability, and simplified chemical processing. However, the preparation and practical application of heterogeneous biocatalysts in both batch and flow processes often encounter challenges, underscoring the need for further investigation and optimization.

For instance, in carrier‐free methods, the formation of cross‐linked enzyme aggregates (CLEAs) often results in particles with low mechanical stability, making them susceptible to breakage or aggregation during processing. This can reduce their effectiveness and complicate handling. Additionally, carrier‐free immobilized enzymes may experience mass transfer limitations. Achieving particle uniformity is also a frequent challenge, leading to inconsistent performance between batches and complicating process scalability.

On the other hand, carrier‐free heterogeneous biocatalysts, including CLEAs, remain highly attractive due to their cost‐effectiveness, as they eliminate the need for a support material. Furthermore, they are typically resistant to enzyme leaching, as cross‐linking is predominantly achieved through covalent bonds, making them particularly well‐suited for flow processes. Continued research and development are therefore essential to enhance the robustness, consistency, and scalability of carrier‐free enzyme immobilization methods.

Interest in entrapment‐based strategies has surged recently, largely due to advancements in 3D printing technologies that facilitate the development of flow bioreactors with embedded enzymes. These innovations enable precise control over the spatial arrangement and distribution of immobilized enzymes, fostering more efficient and customizable biocatalytic systems. However, challenges like mass transfer limitations in (hydro)gels and enzyme leaching from the matrix must still be addressed to optimize immobilized biocatalysts performance.

Given these limitations, heterogeneous systems, where enzymes are physically or chemically bound to a solid support, remain the preferred choice in both batch and flow biocatalysis. Extensive research is focused on improving both the materials and the binding methods used for immobilization. Beyond traditional methacrylate‐ and agarose‐derivatized supports, promising results have been obtained using biopolymers such as lignin and cellulose, as well as specially designed materials like block copolymers and covalent organic frameworks (COFs). Nevertheless, in most cases, further research is required to fully harness the potential of these materials in industrially relevant biotransformations.

When considering enzyme binding to solid supports, covalent binding offers distinct advantages, particularly the prevention of enzyme leaching, which ensures recyclability in batch systems and sustained performance in flow systems. However, a common drawback is the lower recovery of enzyme activity following immobilization. This can be attributed to potential structural alterations in the enzyme during covalent attachment, which may hinder its catalytic function.

On the other hand, non‐covalent immobilization strategies often excel in preserving enzyme activity. Moreover, optimizing non‐covalent immobilization procedures can yield unexpectedly positive outcomes, improving both the stability and overall performance of the biocatalyst. This flexibility makes non‐covalent methods an attractive option for enhancing the efficiency of enzyme immobilization in various applications.

The final section of the review focused on the application of genetic fusion technologies in enzyme immobilization. These techniques, which are widely used in cell biology studies, hold significant potential for enzyme immobilization, particularly when traditional methods prove inadequate. In our view, genetic fusion approaches offer a promising alternative, as they enable the creation of fusion proteins that can be precisely engineered for improved binding, stability, and functionality. This strategy can overcome limitations of simpler methods, paving the way for more efficient and tailored biocatalytic systems.

## Conflict of Interests

The authors declare no conflict of interest.

## Biographical Information

*Daniela Monti is Senior Researcher at the Istituto di Scienze e Tecnologie Chimiche G. Natta (SCITEC), CNR. She received her Master′s degree in Biological Science (1991) and a Specialization degree in Biotechnological Applications (1995) from the University of Milano. Her research activity ranges from the discovery and characterization of novel enzymes to the development of efficient biocatalyzed processes, e. g., through the optimization of enzyme expression in heterologous hosts, the use of free and immobilized enzymes, and the application of multienzymatic and chemo‐enzymatic cascade systems*.